# The effect of Katsura-uri (Japanese pickling melon, *Cucumis melo* var. *conomon*) and its derived ingredient methylthioacetic acid on energy metabolism during aerobic exercise

**DOI:** 10.1186/s40064-015-1144-y

**Published:** 2015-07-26

**Authors:** Wataru Aoi, Kazuya Takeda, Azusa Sasaki, Yuki Hasegawa, Yasushi Nakamura, Eun Young Park, Kenji Sato, Masayo Iwasa, Airi Nakayama, Mizuki Minamikawa, Yukiko Kobayashi, Koji Shirota, Noboru Suetome

**Affiliations:** 1 Laboratory of Health Science, Graduate School of Life and Environmental Sciences, Kyoto Prefectural University, Kyoto, 606-8522 Japan; 2 Laboratory of Food Science, Graduate School of Life and Environmental Sciences, Kyoto Prefectural University, Kyoto, 606-8522 Japan; 3 Division of Applied Biosciences, Graduate School of Agriculture, Kyoto University, Kyoto, 606-8502 Japan; 4 Laboratory of Nutrition Science, Graduate School of Life and Environmental Sciences, Kyoto Prefectural University, Kyoto, 606-8522 Japan; 5 Horticultural Division, Kyoto Prefectural Agriculture, Forestry and Fisheries Technology Center, Kameoka, 621-0806 Japan

**Keywords:** Fragrant ingredient, Aerobic exercise, Skeletal muscle

## Abstract

**Purpose:**

We investigated the effect of Katsura-uri (Japanese pickling melon; *Cucumis melo var. conomon*) on energy metabolism during exercise in human and animal studies.

**Methods:**

Eight healthy men (mean age, 21.4 ± 0.7 years) participated in a single-blind, crossover study. Thirty minutes after ingesting the Katsura-uri drink or placebo drink, they exercised on a cycle ergometer at 40% maximal heart rate for 30 min. Respiratory gas analysis was performed during exercise to examine oxygen consumption and substrate utilization. Blood biochemical parameters were evaluated during exercise. In the animal study, the effect of methylthioacetic acid (MTA), a Katsura-uri derived component was examined in mice. Immediately after running at 25 m/min for 30 min, biochemical parameters in the hind limb muscle and blood of mice were measured.

**Results:**

Oxygen consumption during exercise was higher in the Katsura-uri condition (19.8 ± 3.5 mL/kg/min) than the placebo condition (18.6 ± 3.0 mL/kg/min) (P < 0.05). The elevation of blood lactate was lower in the Katsura-uri condition (1.7 ± 0.4 mM) than the placebo condition (2.2 ± 0.6 mM) 15 min after beginning exercise (P < 0.05). There was a higher positive correlation between lactate concentration and carbohydrate oxidation during exercise in the Katsura-uri condition (R^2^ = 0.86) compared to the placebo condition (R^2^ = 0.47). The decrease in intermuscular pH and the increase in blood lactate following exercise were prevented by MTA supplementation (250 ppm) with significant differences in the MTA-supplemented group compared to the control group.

**Conclusions:**

These results suggest that the ingestion of Katsura-uri and/or MTA improves glucose metabolism and acidification in skeletal muscles during exercise in human and animal studies.

## Background

Kyo-yasai is a general term for heirloom vegetables in Kyoto (Japan) that have been preserved as seeds and grown by using traditional cultivation methods, and some Kyo-yasai have many unique compounds that are not present in conventional vegetables. Katsura-uri (Japanese pickling melon; *Cucumis melo var. conomon*), one of the Kyo-yasai has the unique property of a strong muskmelon-like fragrance (Nakamura et al. 2008); some compounds responsible for the fragrance contribute to its effect on certain physiological functions (antimutagenic, differentiation induction in cancer cells, and antioxidative effects), which are not usually affected by mid-ripened Katsura-uri, or its conventional counterpart (Nakamura et al. 2010). Previously, we identified 6 typical fragrant ingredients of Katsura-uri including methylthioacetic acid ethyl ester (MTAE) (Nakamura et al. 2010), which is acid-hydrolyzed to methylthioacetic acid (MTA) in the stomach. However, an energy metabolism study of whole Katsura-uri fruit ingestion and MTAE/MTA has not yet been reported.

The functional effects of various food factors on energy metabolism during physical exercise have been examined previously (Spriet et al. 1992; Aoi et al. 2008; Murphy et al. 2012; Wall et al. 2011). Exercise requires a large amount of energy to maintain muscle contraction. During exercise, skeletal muscle must continue generating adenosine triphosphate (ATP) by metabolizing nutrients such as carbohydrates and lipids. The capacity of nutrient metabolism is closely associated with exercise-induced muscular fatigue and endurance performance. It is well known that carbohydrate-based energy metabolism via glycolysis results not only in the production of a small amount of ATP but also in muscular acidification by increasing lactic acid production, which leads to impaired muscle contraction. Thus, during aerobic metabolism of carbohydrates in the mitochondria, a large amount of ATP is generated along with a small amount of fatigue-related metabolites. Based on the background regarding possible metabolic regulation of foods, we hypothesized that oral administration of Katsura-uri might improve aerobic metabolism in skeletal muscles, leading to continued muscle contraction. In the present study, we aimed to investigate the effect of Katsura-uri on energy metabolism during exercise and muscular fatigue in humans and to support the results with a follow-up examination using MTA in mice.

## Results

### Indirect metabolic performance in humans

**Fig. 1:**
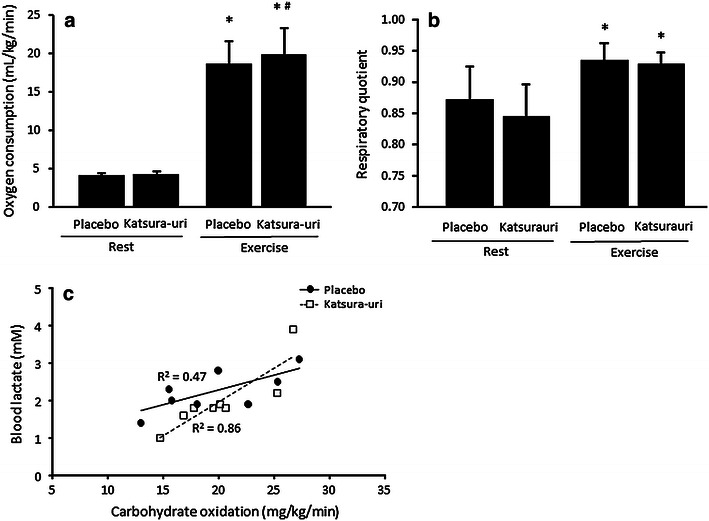
The effect of Katsura-uri drink intake on oxygen consumption (**a**) and respiratory quotient (**b**), and the correlation between carbohydrate oxidation and blood lactate concentration (**c**) in humans. The *continuous line* indicates placebo, and the *dotted line* indicates Katsura-uri. Values are presented as the mean ± SD. *P < 0.05 vs. Rest, ^#^P < 0.05 vs. Placebo.

All subjects reached steady-state exercise intensity between 6 and 12 min after beginning exercise. The mean value of oxygen consumption did not differ between the placebo and Katsura-uri conditions; however, it was significantly higher in the Katsura-uri condition than in the placebo condition during the steady-state period of exercise (P < 0.05) (Fig. 1a). In contrast, the respiratory quotient (RQ) did not differ between the conditions or during the exercise period (Fig. 1b).

There was a significant positive correlation between the lactate concentration after 15 min of exercise and carbohydrate oxidation during the steady-state period of exercise (P < 0.05); in particular, a higher correlation was found in the Katsura-uri condition (R^2^ = 0.86) compared to the placebo condition (R^2^ = 0.47) (Fig. 1c).

### Blood biochemical parameters in humans

**Fig. 2:**
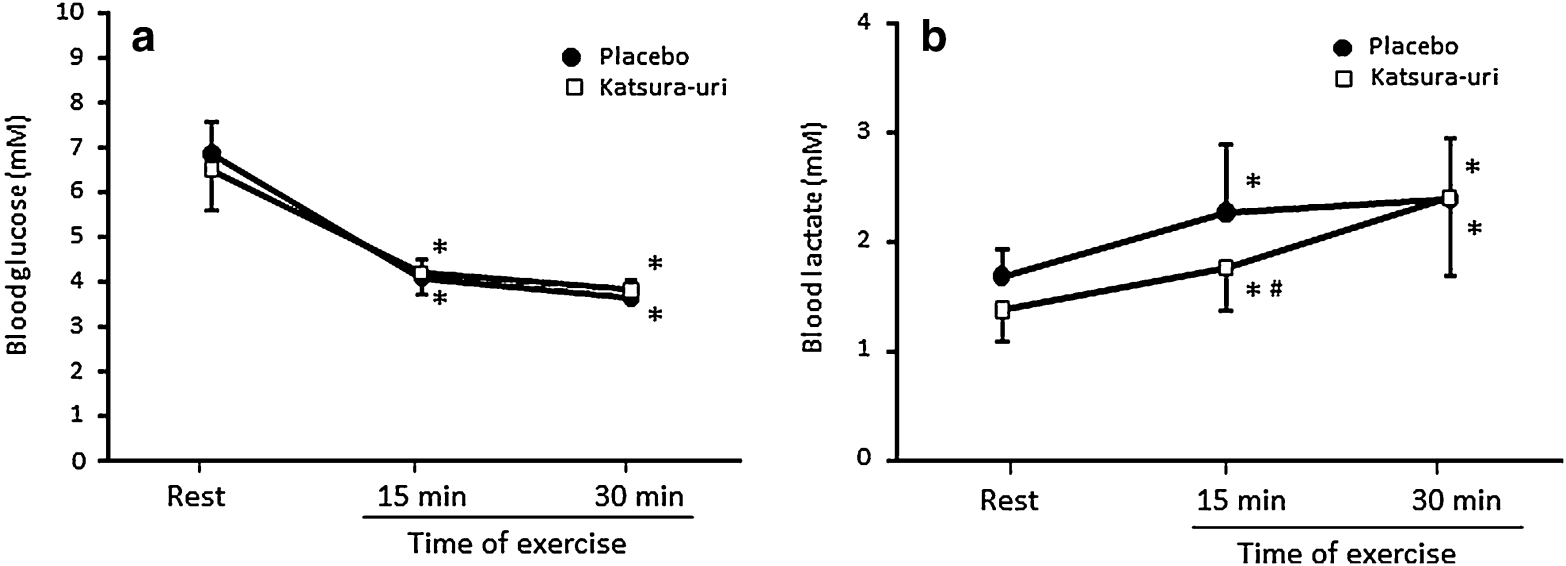
The effect of Katsura-uri drink intake on blood glucose (**a**) and lactate (**b**) concentrations during exercise. Values are presented as the mean ± SD. *P < 0.05 vs. Rest, ^#^P < 0.05 vs. Placebo.

There was a significant decrease in the blood glucose concentrations at 15 and 30 min after exercise, compared to those before exercise in both the placebo and Katsura-uri supplementation conditions (P < 0.05) (Fig. 2a); however, there was no difference in this value between the conditions. Although the blood lactate level was significantly elevated with exercise (P < 0.05), this elevation was significantly lower in the Katsura-uri supplementation condition compared to the placebo condition at 15 min after beginning exercise (mean difference = −0.5, 95% confidence interval = −0.7 to −0.2, P < 0.05) (Fig. 2b).

### Acid-hydrolyzation from MTAE to MTA in artificial gastric juice

**Fig. 3:**
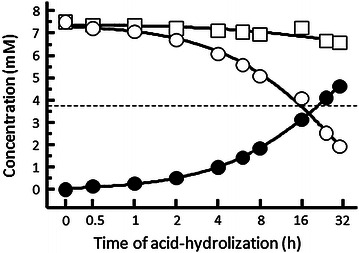
Acid-hydrolyzation from MTAE to MTA in artificial gastric juice. *Unfilled circle* Residual MTAE, *filled circle* MTA formed, *open sqaure* total amount of MTAE and MTA. *Dotted line* expressed 50% of MTAE concentration from the initial concentration at 0 h (7.45 mM). *MTA* methylthioacetic acid, *MTAE* methylthioacetic acid ethyl ester.

The MTAE level began to decline at 30 min in artificial gastric juice, and reached 50% of the concentration between 16 and 24 h (Fig. 3). Simultaneously, MTA was formed 30 min after incubation in artificial gastric juice.

### Blood parameters in mice

There were no significant differences in blood glucose and plasma non-esterified fatty acid (NEFA) levels between the control and MTA groups either at rest or during exercise (Table 1). In contrast, blood ammonia levels after exercise showed a tendency to decrease in a dose-dependent manner in the MTA supplementation group compared to that in the control group (Table 1). Although blood lactate level was significantly higher with exercise (P < 0.05), this elevation was suppressed by the administration of 250 ppm of MTA (P < 0.05) (Table 1).

**Table 1:** **Blood metabolic parameters in mice**

	Sedentary	Exercise		
		Control	MTA-25	MTA-250
Blood glucose (mg/dL)	6.6 ± 0.7	7.8 ± 1.4	7.5 ± 2.0	6.7 ± 1.0
Plasma NEFA (mM)	880 ± 281	916 ± 360	883 ± 529	889 ± 268
Blood ammonia (μM)	110 ± 43	135 ± 37	125 ± 68	107 ± 33
Blood lactate (mM)	**–**	3.2 ± 1.2	2.5 ± 0.7	2.1 ± 0.3^#^

Values are presented as mean ± SD. Control, exercise group administered water.

*MTA-25* exercise group receiving 25 ppm MTA supplementation, *MTA-250* exercise group receiving 250 ppm MTA supplementation, *MTA* methylthioacetic acid.

^#^P < 0.05 vs. control.

### Intermuscular pH in mice

**Fig. 4:**
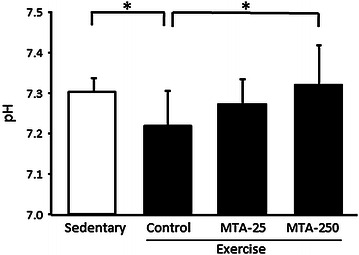
The effect of MTA on intermuscular pH in mice. *Control* control-administered exercise group, *MTA-25* 25 ppm MTA-administered exercise group, *MTA-250* 250 ppm MTA-administered exercise group, *MTA*, methylthioacetic acid. Values are presented as the mean ± SD. *P < 0.05.

The interstitial pH levels in muscle significantly reduced following exercise (P < 0.05). However, this reduction was suppressed by the administration of MTA, with a significant difference between the control and MTA supplementation group of 250 ppm (P < 0.05, Fig. 4).

### Glycogen and enzyme activity in mouse muscle

Muscle glycogen showed a tendency to decrease with exercise, and there was a significant decrease in muscle glycogen in the group with 250 ppm MTA supplementation (P < 0.05) (Table 2). Succinate dehydrogenase (SDH), an enzyme of the Krebs cycle, showed a tendency to increase with MTA in a dose-dependent manner. Both carnitine palmitoyltransferase Ι (CPTI), a rate-limiting enzyme of fatty acid entry into the mitochondria, and pyruvate dehydrogenase (PDH), an enzyme that links the glycolysis pathway to the Krebs cycle, were unchanged on either exercise or MTA supplementation (Table 2).

**Table 2:** Muscle metabolic parameters in mice

	Sedentary	Exercise		
		Control	MTA-25	MTA-250
Glycogen content (μg/g)	40.0 ± 11.0	33.7 ± 7.5	29.9 ± 11.3	26.4 ± 7.8*
CPTI activity (nmol/mg prot.)	4.9 ± 1.0	5.2 ± 0.9	4.9 ± 1.3	5.4 ± 0.9
SDH activity (μmol/mg prot.)	27.4 ± 15.2	21.3 ± 14.9	27.9 ± 13.4	29.4 ± 23.2
PDH activity (OD change/15 min)	0.067 ± 0.026	0.078 ± 0.003	0.068 ± 0.024	0.066 ± 0.035

Values are presented as mean ± SD.

*Control* exercise group administered water, *MTA-25* exercise group receiving 25 ppm MTA supplementation, *MTA-250* exercise group receiving 250 ppm MTA supplementation, *MTA* methylthioacetic acid, *CPTI *carnitine palmitoyltransferase Ι, *SDH* succinate dehydrogenase, *PDH* pyruvate dehydrogenase.

* P < 0.05 vs. sedentary.

## Discussion

In the present study, the intake of Katsura-uri resulted in increased oxygen consumption and lower levels of blood lactate in humans, and a higher correlation was observed between blood lactate levels and carbohydrate oxidation during exercise in the Katsura-uri condition than in the placebo condition. Similarly, the administration of the Katsura-uri-derived compound formed in the stomach, MTA, suppressed both blood lactate elevation and intramuscular pH reduction in response to exercise in mice. Although Katsura-uri contains several fragrant compounds, including MTAE (the precursor of MTA), the effects of the intact fruit or its constituent compounds on energy metabolism have not been completely elucidated. To our knowledge, the present study is the first to demonstrate that both Katsura-uri and MTA can improve aerobic metabolism during prolonged exercise, thus leading to reduced exercise-induced muscle acidification. Although MTA is not present in the whole Katsura-uri fruit, it is formed by the acid-hydrolyzation of MTAE in stomach after oral intake of the fruit; our observations suggest that the effect of Katsura-uri might be associated with MTA formation in the stomach.

During muscle contraction, lactic acid, a major source of protons, is rapidly produced by glycolytic metabolism, thus lowering pH and preventing muscle contraction. The results of both the human and animal studies indicate that the blood lactate concentration following exercise is lowered by the intake of either Katsura-uri or MTA drink. In the animal study, the difference in interstitial pH between the placebo and MTA supplementation groups was directly demonstrated. The protons dissociated from lactic acid are immediately buffered in the cytosol or exported to the interstitial fluid and further transported to circulation. The buffering capacity is relatively higher in cytosol and blood, whereas this capacity is lower in interstitial fluid because it contains fewer buffering factors (Fogh-Andersen et al. 1995; Aukland and Fadnes 1973). Therefore, the pH of the interstitial fluid in muscle tissues changes easily in response to muscle contraction. Our results suggest that both Katsura-uri drink and MTA supplementations prevent muscle acidification by inhibiting the generation of lactic acids, and that this might result in less fatigue and contribute to continued muscle contraction. The decrease in lactic acid production generally results from a decrease in activity of the glycolytic pathway or from the accelerated entry of pyruvate into the mitochondria, where it is metabolized aerobically. The glycogen content in the muscle after exercise was lower in the MTA group than in the control group in the animal study, suggesting that an improvement in aerobic metabolism might lead to a decrease in lactic acid production.

We found a higher correlation between blood lactate concentration and carbohydrate oxidation in the Katsura-uri group compared to that in the placebo group. Although both parameters depend on the degree of glucose metabolism, lactate is the end product of the glycolytic pathway, and carbohydrate oxidation is indicative of aerobic metabolism of glucose in the mitochondria. Therefore, this result supports the hypothesis that Katsura-uri can accelerate aerobic glucose metabolism, which might lead to prevention of muscle acidification by suppressing lactic acid production.

It is well known that oxygen consumption is strongly correlated with energy expenditure. In the human study, we found higher oxygen consumption in the Katsura-uri group than in the placebo group during exercise, but not at rest, indicating that the Katsura-uri drink elevated energy expenditure only during exercise. However, in this study, we did not here examine which factors contributed to the increase of oxygen consumption due to Katsura-uri intake, which was a limitation of this study. Because the mechanical work load to maintain exercise was equal between the two groups, accelerating thermogenesis might have caused the increase in energy expenditure with consumption of the Katsura-uri drink. Previously, several phytochemicals have been shown to increase energy metabolism through thermogenesis in metabolic tissues in the sedentary state (Yoneshiro et al. 2012; McCarthy et al. 2011; Lee et al. 2011), but their such effects on metabolism during exercise have not yet been reported. Future studies are needed to examine the mechanism of accelerated energy expenditure during exercise.

A limitation of this study is that we did not specifically clarify the mechanism of action of Katsura-uri. Both Katsura-uri drink and MTA were found to have certain effects on energy metabolism despite the fact that they were ingested only 30 min before exercise. We observed the formation of MTA at 30 min after the start of MTAE-incubation in artificial gastric juice, and this result supports the hypothesis that MTA formed from MTAE in the stomach can be rapidly absorbed, circulated, and can then exerted its function in the skeletal muscle. Future studies should clarify the retention time of MTAE in the stomach when humans ingest Katsura-uri, and the amount of MTA formed in the stomach or other organs. In addition, it is unlikely that the compound exerted its function through transcriptional and translational mechanisms in such a short time period. Therefore, we propose that MTA might activate muscular enzymes that play important roles in maintaining aerobic metabolism (Costill et al. 1976; Holloway et al. 2006; Spriet and Heigenhauser 2002). However, we could not find any significant change in the levels of several key enzymes, although SDH, a rate-limiting enzyme of the Krebs cycle, showed a tendency to increase following MTA supplementation. Thus, further studies are needed to determine the specific mechanism by which MTA affects muscle metabolism and as well as to determine the optimal timing and amount of Katsura-uri ingestion based on its characteristics, because clarification of these issues was limited in the present study. Moreover, the human study showing the effect of Katsura-uri ingestion was a pilot study with a small number of participants and a limited protocol; it was specifically designed to be supported by the mouse model study to show the effect of MTA in mice. Further research is required to examine whether these observations can be generalized in a larger sample size; in addition, further investigation is warranted to identify whether any ergogenic effect is possible for athletes.

## Conclusion

Results of the primary human study showed that the intake of Katsura-uri before exercise suppressed the elevation of blood lactate levels, and that the intake was correlated with higher oxygen consumption during exercise. In the animal study, the administration of the Katsura-uri-derived compound MTA to mice also resulted in the suppression of elevated lactate levels and reduced intramuscular pH. These observations suggest that Katsura-uri can bring some advantage in athletic fields due to improvement of glucose aerobic metabolism in skeletal muscle during exercise, and a part of this process might be mediated by MTA.

## Methods

### Human subjects

Eight healthy men (mean age, 21.4 ± 0.7 years; height, 172.3 ± 7.1 cm; body weight, 63.7 ± 4.9 kg; body mass index, 21.5 ± 2.1 kg/m^2^) were recruited as participants in a single-blind, crossover study, which was approved by the ethics committee of Kyoto Prefectural University (No. 45). All the subjects provided written informed consent. No subject had a current or prior chronic disease or a history of smoking, and no subject was currently using any medication. Moreover, no subject was habituated to a regular exercise regimen.

### Test drink

Katsura-uri was cultivated and harvested after it was ripened in 2012–2013 in an open field culture system at the Kyoto Prefectural Agricultural Research Institute (Kameoka, Japan). After removal of the peels and seeds, the residual pieces were vacuum-packed with a plastic bag and stored at −25°C until ready for use in the experiment. Katsura-uri drink was prepared by the following method: 500 g of frozen Katsura-uri fruit was partially thawed, chopped, and mixed by using an electric automatic mixer with 10.6 g of a sweetener (Pal Sweet; Ajinomoto, Tokyo, Japan). The placebo drink was made by dissolving 6.0 g fructose, 5.5 g glucose, and 2.5 g sucrose in 484 g water, while adjusting for the equivalent composition and amount of carbohydrates contained in Katsura-uri (unpublished data), and adding 10.6 g Pal Sweet. Subjects were not informed about the characteristics of Katsura-uri, which limited their ability to distinguish between active and placebo drinks and removed bias. Thus, this study was performed under a single-blind condition. The Katsura-uri drink and the placebo were prepared on the day before each trial, and administered to subjects by pouring them into identical cups; therefore, the outward appearance of the cup did not distinguish between Katsura-uri and placebo drinks.

### Human experiment design

The subjects were asked to fast, except for water consumption, from 22:00 the night prior to the experiment. On the experiment day, all subjects consumed the same breakfast (200 g of steamed rice and 170 ml of miso soup [Asage; Nagatanien, Tokyo, Japan]) at 8:30 to normalize the effects of a pre-exercise meal. Thirty minutes after drinking the Katsura-uri drink or the placebo beverage at 10:00, all participants performed a single session of steady-state cycling exercise for 30 min. The work load was gradually increased by 10 W every 2 min beginning with 50 W until the heart rate reached the predicted 40% of the maximum heart rate, which determined by using the Karvonen formula (Karvonen 1957), and this work load was maintained until the end of exercise. The measurement of respiratory gas was initiated 15 min before exercise and continued until after exercise. Blood glucose and lactate levels were measured at rest and after 15 and 30 min of beginning exercise by using a simple finger stick blood test (Lactate Pro, Glu Test; Arkray, Kyoto, Japan). There was a 1-week washout period prior to the test, and all the subjects completed both testing conditions.

### Indirect metabolic performance

Respiratory gas oxygen consumption (VO_2_) and carbon dioxide production (VCO_2_) were measured using a breath-by-breath respirometer system (Aeromonitor AE310S; Minato, Osaka, Japan). To reduce breath-by-breath variation, these data were analyzed using a mean value obtained every 60 s. The RQ and substrate utilization were calculated from the level of VO_2_ and VCO_2_, as described previously (Frayn 1983).

### Mice experimental design

This study complied with the guidelines of the Japanese Council on Animal Care, and it was approved by the Committee for Animal Research of the University (M24-49). ICR mice (7-week-old; Shimizu Laboratory Supplies, Kyoto, Japan) were acclimatized for 1 week in an air-conditioned (22 ± 2°C) room on a 12-h light/dark cycle (lights on from 7:30 to 19:30). Mice were divided into the following 4 groups with 10 mice in each group: sedentary, exercise with control, exercise with supplementation of 25 ppm MTA, and exercise with supplementation of 250 ppm MTA.

MTA solutions were prepared with water to suitable concentration and given to mice 30 min before exercise (10 μL/g body weight). Water was provided to control mice in the same volume as MTA supplementation mice. After the oral administration, mice in the exercise groups ran on a treadmill at 25 m/min for 30 min. Immediately after exercise, intermuscular pH was measured under anesthesia, and blood concentrations of glucose and lactate were obtained and measured (GluTest, LactatePro; Arkray) from the tail vein. The gastrocnemius muscles and blood via cardiocentesis were collected. The sedentary mice underwent the same blood and muscle collection procedures. Blood ammonia and plasma NEFA levels were measured using assay kits, respectively (Wako, Osaka, Japan).

### Glycogen content in skeletal muscle

Muscle tissues were homogenized in 0.3 M hypochlorous acid, incubated with amyloglucosidase (25 mg protein/6 mL) and 20 mM sodium acetate, and quantified using a d-glucose measurement kit (F-kit, Roche Diagnostics, Basel, Switzerland).

### Enzyme activity in skeletal muscle

Enzyme activity was measured using muscle homogenates. SDH and CPTI levels were measured according to previously described protocols (Cooperstein et al. 1950; Bieber et al. 1972). PDH activity was measured using a commercial enzyme-linked sorbent assay kit (Abcam, Cambridge, UK), and data was expressed as OD change at 450 nm for 15 min of enzymatic reaction.

### Acid-hydrolyzation from MTAE to MTA in artificial gastric juice

Five milligrams of MTAE (Alfa Aesar, Lancashire, UK) was dissolved with 5.0 mL of artificial gastric juice (actual concentration of MTAE: 1 mg/mL or 7.45 mM). The tube was incubated for 0.5–30 h at 37°C, a 200 μL aliquot of the solution was withdrawn at several time points, and residual MTAE and MTA (Matrix Scientific, Columbia, SC, USA) formed in the artificial gastric juice were measured by using reverse-phase HPLC–UV. The levels of MTAE and MTA was measured using an HPLC model LC-20AT with a SPD-20A UV detector (Shimadzu, Kyoto, Japan). A YMC-pack Pro C18-RS (Ø4.6 × 150 mm) analytical column was used for MTAE and MTA analyses based on reverse phase chromatography. A 10 μL aliquot of sample was injected and isocratically eluted using 18% acetonitrile in 0.1% trifluoroacetate mobile phase. The UV absorbance at 230 nm was used to detect MTA at 3.5 min and MTAE at 23.5 min.

### Statistical analysis

All data are presented as the mean ± SD. Differences between groups were evaluated using a two-way analysis of variance. When significant interactions were detected, post hoc multiple comparisons were conducted using the Bonferroni method. When data were not normally distributed, differences between groups were tested using non-parametric tests. To further assess time course comparisons, the mean differences in blood parameter changes between conditions or time points as well as the 95% confidence intervals were calculated. The correlation between substrate oxidation and lactate concentration was evaluated by performing Spearman’s correlation analysis. P < 0.05 was considered statistically significant.

## Abbreviations

ATP: adenosine triphosphate
MTA: methylthioacetic acid
MTAE: methylthioacetic acid ethyl ester
RQ: respiratory quotient
NEFA: non-esterified fatty acid
SDH: succinate dehydrogenase
CPTI: carnitine palmitoyltransferase Ι
PDH: pyruvate dehydrogenase
