# Erratum to: The effect of Katsura-uri (Japanese pickling melon, *Cucumis melo* var. *conomon*) and its derived ingredient methylthioacetic acid on energy metabolism during aerobic exercise

**DOI:** 10.1186/s40064-015-1305-z

**Published:** 2015-09-21

**Authors:** Wataru Aoi, Kazuya Takeda, Azusa Sasaki, Yuki Hasegawa, Yasushi Nakamura, Eun Young Park, Kenji Sato, Masayo Iwasa, Airi Nakayama, Mizuki Minamikawa, Yukiko Kobayashi, Koji Shirota, Noboru Suetome

**Affiliations:** Laboratory of Health Science, Graduate School of Life and Environmental Sciences, Kyoto Prefectural University, Kyoto, 606-8522 Japan; Laboratory of Food Science, Graduate School of Life and Environmental Sciences, Kyoto Prefectural University, Kyoto, 606-8522 Japan; Division of Applied Biosciences, Graduate School of Agriculture, Kyoto University, Kyoto, 606-8502 Japan; Laboratory of Nutrition Science, Graduate School of Life and Environmental Sciences, Kyoto Prefectural University, Kyoto, 606-8522 Japan; Horticultural Division, Kyoto Prefectural Agriculture, Forestry and Fisheries Technology Center, Kameoka, 621-0806 Japan

## Erratum to: SpringerPlus (2015) 4:377 DOI 10.1186/s40064-015-1144-y

It has come to our attention that during production of the original article, an error was introduced into Table 1 during copyediting. The corrected Table 1 can be found below. The publisher apologises for inconvenience caused.

**Table 1:** Blood metabolic parameters in mice

	Sedentary	Exercise		
		Control	MTA-25	MTA-250
Blood glucose (mM)	6.6 ± 0.7	7.8 ± 1.4	7.5 ± 2.0	6.7 ± 1.0
Plasma NEFA (μEq/L)	880 ± 281	916 ± 360	883 ± 529	889 ± 268
Blood ammonia (μM)	110 ± 43	135 ± 37	125 ± 68	107 ± 33
Blood lactate (mM)	–	3.2 ± 1.2	2.5 ± 0.7	2.1 ± 0.3^#^

Values are presented as mean ± SD. Control, exercise group administered water; MTA-25, exercise group receiving 25 ppm MTA supplementation; MTA-250, exercise group receiving 250 ppm MTA supplementation

*MTA* methylthioacetic acid

^#^P < 0.05 vs. control

